# Associations of lipids and lipid-modifying drug target genes with atrial fibrillation risk based on genomic data

**DOI:** 10.1186/s12944-024-02163-4

**Published:** 2024-06-08

**Authors:** Yuhang Tao, Yuxing Wang, Yongkun Yin, Kai Zhang, Yingchao Gong, Hangying Ying, Ruhong Jiang

**Affiliations:** Department of Cardiology, Sir Run Run Shaw Hospital, School of Medicine, Zhejiang University, 3 East Qingchun Road, Hangzhou, Zhejiang, 310016 P.R. China

**Keywords:** Lipids, Drug target, Atrial fibrillation, Mendelian randomization

## Abstract

**Background:**

The causal associations of lipids and the drug target genes with atrial fibrillation (AF) risk remain obscure. We aimed to investigate the causal associations using genetic evidence.

**Methods:**

Mendelian randomization (MR) analyses were conducted using summary-level genome-wide association studies (GWASs) in European and East Asian populations. Lipid profiles (low-density lipoprotein cholesterol, triglyceride, and lipoprotein[a]) and lipid-modifying drug target genes (3-hydroxy-3-methylglutaryl-CoA reductase, proprotein convertase subtilisin/kexin type 9, NPC1-like intracellular cholesterol transporter 1, apolipoprotein C3, angiopoietin-like 3, and lipoprotein[a]) were used as exposures. AF was used as an outcome. The inverse variance weighted method was applied as the primary method. Summary-data-based Mendelian randomization analyses were performed for further validation using expression quantitative trait loci data. Mediation analyses were conducted to explore the indirect effect of coronary heart disease.

**Results:**

In the European population, MR analyses demonstrated that elevated levels of lipoprotein(a) increased AF risk. Moreover, analyses focusing on drug targets revealed that the genetically proxied target gene *LPA*, which simulates the effects of drug intervention by reducing lipoprotein(a), exhibited an association with AF risk. This association was validated in independent datasets. There were no consistent and significant associations observed for other traits when analyzed in different datasets. This finding was also corroborated by Summary-data-based Mendelian randomization analyses between *LPA* and AF. Mediation analyses revealed that coronary heart disease plays a mediating role in this association. However, in the East Asian population, no statistically significant evidence was observed to support these associations.

**Conclusions:**

This study provided genetic evidence that Lp(a) may be a causal factor for AF and that *LPA* may represent a promising pharmacological target for preventing AF in the European population.

**Supplementary Information:**

The online version contains supplementary material available at 10.1186/s12944-024-02163-4.

## Introduction

Globally, millions of people suffer from atrial fibrillation (AF), one of the most common cardiac arrhythmias [1]. Due to longer lifespans, it is expected that the prevalence of AF will continue to rise, and the related social and medical burdens are growing. An increasing number of studies have confirmed that AF may increase the risk of stroke, heart failure, dementia, and early mortality [2]. However, little is known about its pathology, and no treatment is currently effective in reversing the progression of AF. Therefore, the identification of controllable risk factors for AF is a high priority for prevention.

Dyslipidemia is a well-established risk factor for numerous cardiovascular diseases, and may indeed serve as a contributing factor to the burden of AF. Indispensable to primary health care, cardiovascular medications, including lipid-lowering agents, play a pivotal role in promoting overall well-being [3]. However, the associations with the risk of AF remain ambiguous [4–8]. In light of the increasing prevalence of AF, assessing the impacts of lipids and their respective pharmacotherapies on this condition of great significance.

However, given the nature of observational studies, unidentified factors may confound the results, making the causality of the associations contradictory. Randomized controlled trials (RCTs) are recognized to overcome the limitations of observational studies. However, RCTs are notoriously difficult to implement because of ethical and economic concerns.

Genetic epidemiology offers an alternative approach to tackle these inquiries. The genetic variants found within or in proximity to target genes can impact protein expression or function. These genetic effects can then be leveraged to predict the potential outcomes of pharmacological action [9]. Drug target Mendelian randomization (MR) is a genetic statistical approach, that evaluates the causal effect of genetically proxied drug targets on the clinical outcome of interest. This is accomplished by applying genetic variants in genes encoding drug targets as instrumental variables (IVs) [10]. The majority of genetic variants, such as single nucleotide polymorphisms (SNPs), are identified through genome-wide association studies (GWASs) that are publicly available. By virtue of the stochastic allocation of genetic material from parents to their progeny during conception, MR can minimize confounding and reverse causality biases [11].

In this study, we utilized MR methodology to investigate causal associations in European and East Asian populations. Initially, we performed two-sample MR analyses to determine the causal role of lipids in the susceptibility to AF. Subsequently, we conducted MR investigations of drug targets to examine the effects of genetic proxies of drug targets on the risk of AF. Finally, we conducted a sequence of validation analyses to attain results of utmost reliability and validity.

## Materials and methods

**Fig. 1:**
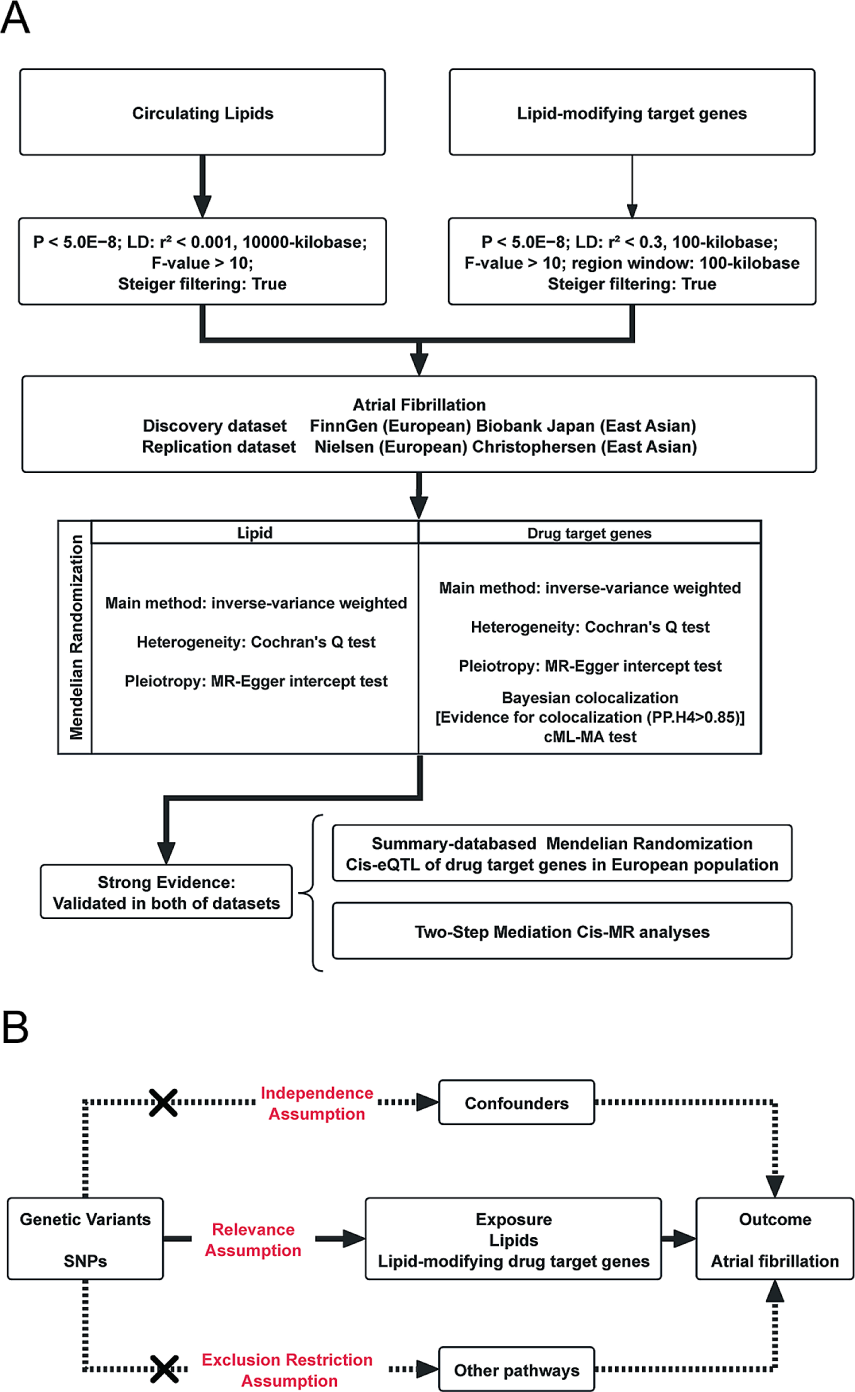
Flowchart of this study and MR assumptions

Our research conformed to the principles and criteria of the reporting guidelines of the Strengthening the Reporting of Observational Studies in Epidemiology-Mendelian Randomization (STROBE-MR) (Table S1). A comprehensive illustration of the study design is shown in Fig. 1A. To obtain the required data, we used publicly available summary-level data from GWASs, as well as studies on expression quantitative trait loci (eQTLs). Detailed information concerning these datasets is provided in Table S2.

### Genetic proxies for exposures

The MR approach was based on three predominant assumptions: (1) genetic variants are strongly associated with exposure; (2) there are no genetic variants related to any possible confounders; and (3) there are no genetic variants related to AF except via exposure (Fig. 1B).

We identified IVs by selecting all SNPs linked to low-density lipoprotein cholesterol (LDL-C), triglyceride (TG), and lipoprotein(a) (Lp[a]) at a genome-wide significance threshold (*P <* 5.0 × 10^− 8^). These IVs were chosen without considering the genomic locations of SNPs. The GWASs of LDL-C and TG used in this study were obtained from the Global Lipids Genetics Consortium [12]. The Neale Laboratory [13] and Pan-UKB team [14] provided summary-level Lp(a) data from the UK Biobank.

To guarantee the independence of IVs, the identified genetic variants underwent a filtration process based on their linkage disequilibrium (LD), assessed by means of a correlation coefficient measurement, denoted as r² (with the criterion of r² < 0.001 within a 10,000-kilobase window, using the European or East Asian reference panel derived from the 1000 Genomes Project) [15].

For the drug target MR analyses, in accordance with the pharmacological mechanism, the genes encoding the targets were identified using DrugBank and previous studies. 3-Hydroxy-3-methylglutaryl-CoA reductase (*HMGCR*), proprotein convertase subtilisin/kexin type 9 (*PCSK9*), NPC1 like intracellular cholesterol transporter 1 (*NPC1L1*), apolipoprotein C3 (*APOC3*), angiopoietin like 3 (*ANGPTL3*), and lipoprotein(a) (*LPA*) were chosen as lipid-modifying drug target genes. The locations of the target genes were checked at the National Center for Biotechnology Information (accessed on 5 June, 2023). This information is listed in Table S3.

To mimic the effect of the target genes, we discerned IVs through the comprehensive selection of all SNPs situated within the 100-kilobase window surrounding the locus of the target genes, which exhibited robust associations with lipids at a significance threshold (*P <* 5.0 × 10^− 8^). Furthermore, the identified genetic variants were subjected to a clumping procedure, ensuring a pairwise correlation coefficient (r²) of less than 0.30 within the 100-kilobase window.

In consideration of confounders, we used the LDtrait tool to identify SNPs that were possibly related to confounding factors (blood pressure, body mass index, type 2 diabetes and so on). Relevant SNPs were removed from the subsequent analyses.

In addition, eQTL variants were used for further study in the European population. We utilized publicly accessible data sourced from the Genotype-Tissue Expression (GTEx) Consortium Version 8 [16]. The cis-QTL variants were characterized by their location within a range of 1 megabase, either upstream or downstream of the transcription start site of the gene responsible for encoding proteins.

### Genetic association for outcomes

In the case of European populations, summary data of the discovery dataset for AF were derived from the FinnGen Release 10 [17]. Cases of AF were discerned through diagnostic coding based on the 8th, 9th, and 10th revisions of the International Classification of Diseases (ICD). FinnGen (https://www.finngen.fi/) is designed to combine genomic information and health information to improve human wellness. For the replication analysis, we used the GWAS data from Nielsen’s study, encompassing the most recent meta-analysis of GWASs of six distinct cohorts [18]. In the case of the East Asian population, we conducted discovery analyses using GWASs of Asians from the Biobank Japan [19], and replication analyses were performed using summary GWASs from the Ingrid E Christophersen’s study [20]. All the information is listed in Table S2.

To corroborate the validity of our chosen genetic variants as drug targets, we conducted a positive control analysis. The primary indication for lipid-modifying drugs was CHD, which was regarded as the positive control outcome. The GWASs were retrieved from the CARDIo-GRAMplusC4D consortium [21] and Biobank Japan [19].

In all cases, the GWASs were conducted in European or East Asian ancestry populations with relevant ethical approval and participant consent obtained. Therefore, the ethical approval process did not require any additional steps for this study.

### Statistical analysis

First, two-sample MR analyses were conducted to investigate the causal relationships. The Steiger filtering approach was utilized to include SNPs with the correct causal direction [22].

To assess whether the retained SNPs may suffer from weak instrument bias, we calculated the F value of the IVs, considering an F value greater than 10 as indicative of strong instrument strength [23]. It was calculated using the following formula [24]: $\text{F}=\frac{{\text{R}}^{2}\times (\text{N}-2)}{1-{\text{R}}^{2}}$, ${\text{R}}^{2}=\frac{2\times \text{E}\text{A}\text{F}\times (1-\text{E}\text{A}\text{F})\times {\text{b}\text{e}\text{t}\text{a}}^{2}}{2\times \text{E}\text{A}\text{F}\times \left(1-\text{E}\text{A}\text{F}\right)\times {\text{b}\text{e}\text{t}\text{a}}^{2}+2\times \text{E}\text{A}\text{F}\times (1-\text{E}\text{A}\text{F})\times \text{N}\times {\text{S}\text{E}}^{2}}$, where R² signifies the proportion of the variance in lipids elucidated by each IV, N denotes the sample size of the GWAS conducted for lipids, EAF represents the frequency of the effect allele, beta embodies the estimated genetic effect, and SE characterizes the standard error of the genetic effect. To ensure that our study had adequate statistical power, we utilized the online tool mRnd [25] (http://cnsgenomics.com/shiny/mRnd/) to perform the necessary calculations.

The inverse variance weighted (IVW) method served as the principal analytical strategy in our analyses. All estimates (odds ratios [ORs]) for AF risk reflected the equivalent of a 1-unit change in lipid concentrations (mmol/L for LDL-C and TG, nmol/L for Lp[a]). The selection between a fixed or random-effects model was determined based on the level of heterogeneity observed within the data. The Cochran’s Q test was utilized to evaluate the observed heterogeneity [26]. If the *P* value of the Cochran’s Q test was less than 0.05, indicating the presence of heterogeneity, the final results of the MR study were obtained by adopting the outcome derived from the multiplicative random effects IVW method. However, if the *P* value of Cochran’s Q test was equal to or greater than 0.05, suggesting no significant heterogeneity, the fixed effects IVW method was used as the primary approach to determine the final results. The weighted median [27], maximum likelihood [28] and MR‒Egger methods were further applied as additional analyses for evaluating causal relationships. If only a single SNP remained, the causality was assessed via the Wald ratio method. The analyses were replicated in another dataset for external validation. We also applied meta-analysis to combine the effect estimates from both the discovery and validation results of the IVW method as supplemental results [29]. The confirmation of significance in all outcomes was considered the definitive affirmation of a statistically significant conclusion.

Egger regression intercepts [30] were used to identify the potential horizontal pleiotropy. If the intercepts of the MR‒Egger regression exhibited a significant deviation from zero with a *P* value less than 0.05, it was regarded as proof of pleiotropic bias, and the outcomes of the MR‒Egger method were considered conclusive. To evaluate whether a SNP had a disproportionate influence on the overall estimates, a leave-one-out sensitivity analysis was conducted. This analysis involved iteratively removing each SNP from the model and examining the resulting impact on the estimated effects.

Drug target MR analysis is referred to as cis-MR analysis [31], which is a specific type of MR that uses variants originating from a single gene region. Adjacent genetic variants are often inherited together, creating a correlation known as LD. This correlation may also contribute to the potential biases in the results. Bayesian colocalization analysis was subsequently conducted to determine the possibility of LD. It was utilized with the prior probabilities set at 10^− 4^ for each variant being the causal variant for the exposure trait, 10^− 4^ for the outcome trait and 10^− 5^ for both traits. The Bayesian colocalization analysis generated some informative outputs: posterior probability for H0, neither trait has a genetic association in the region; H1, only the exposure trait has a genetic association in the region; H2, only the outcome trait has a genetic association in the region; H3, both traits are associated, but with different causal variants; H4, both traits are associated and share a single causal variant. A posterior probability exceeding 0.85 indicated strong evidence of colocalization. This analysis predominantly utilized the coloc (version 5.2.2) R package, and the “abf” method was used as its primary approach.

However, the affirmative findings from the colocalization analysis suggest a shared set of genetic causal variants between the exposure and the outcome, distinctly indicating the presence of horizontal pleiotropy [32]. An MR method named cML-MA (constrained maximum likelihood and model averaging) has been formulated to effectively regulate both correlated and uncorrelated pleiotropic effects [33]. It contains two main approaches: cML-MA-BIC (constrained maximum likelihood-model averaging-Bayesian information criterion) and cML-MA-BIC-DP (constrained maximum likelihood-model averaging- Bayesian information criterion-data perturbation). Goodness-of-fit tests were conducted to determine which option is most suitable. If the test result was deemed significant, then the recommended approach is to apply cML-MA-BIC-DP; otherwise, cML-MA-BIC is the preferred choice. Hence, the cML-MA method was used to further substantiate the findings.

### Supplementary analyses

To validate the reliability of our findings, we conducted a comprehensive set of supplementary analyses for the drug targets with significant associations. We conducted drug target MR analyses using more stringent LD thresholds, specifically r² < 0.1, r² < 0.01, and r² < 0.001, to investigate the outcomes. Then, we used cis-eQTL variants for further study to sharpen the strength of the previous conclusions. We conducted summary-data-based Mendelian randomization (SMR) to explore associations [34]. The analyses utilized summary data from both GWASs and eQTL studies in European populations [35]. Additionally, the heterogeneity in dependent instruments (HEIDI) test was performed to distinguish pleiotropy from linkage, where a *P* value < 0.01 indicated that the associations were likely attributed to high LD [36].

In addition, considering that CHD serves as a predisposing factors for AF, it may function as an intermediary to elucidate the impact of lipid-modifying drug target genes on susceptibility to AF. We conducted mediation analyses and included the risk factor in the model to explore their possible mediating effect using the discovery dataset. We used the “Two-Step Cis-MR” approach [37], which alleviated bias resulting from the strong LD in cis-MR analysis, to evaluate the direct effect of lipid-modifying genes on AF after adjusting for the effect of CHD. To assess the mediation effect, two-step MR analyses were employed. The first step involved evaluating the effect of lipid-altering genes on CHD, referred to here as β1. The subsequent step explored the effect of CHD on AF, denoted as β2. Using the “product of coefficients” approach, we calculated the indirect effect as the product of β1 and β2 [37]. The standard errors for the indirect effect were determined by delta method [38]. Besides, to further determine whether genetic predispositions for *LPA* and CHD were causal genetic risk factors independent of one another, we conditioned the summary GWAS of AF on the summary GWAS of CHD using multi-trait conditional and joint analysis (mtCOJO) [39].

Multiple testing corrections were applied with a significance threshold set as the Bonferroni corrected *P* value. For analyses between lipids and AF risk, a *P* value < 4.17 × 10^− 3^ (0.05/12) was used to adjust for multiple testing of 3 lipid species, 2 AF datasets and 2 ancestries. For analyses between lipid-modifying genes and AF risk, a *P* value < 2.08 × 10^− 3^ (0.05/24) was used to adjust for multiple testing of 6 lipid-modifying genes, 2 AF datasets and 2 ancestries. For other analyses, a *P* value < 0.05 was considered to indicate statistical significance. The analyses were executed within the R environment, leveraging specialized packages including MendelianRandomization [40] (version 0.9.0), TwoSampleMR [41] (version 0.5.6), TwoStepCisMR [37] and coloc [42] (version 5.2.2). The forest plots were drawn with the forestploter package. SMR and HEIDI tests were performed using SMR software [35] (version 1.3.1). MTCOJO analysis was conducted using GCTA software [39] (version 1.94.1).

## Results

### Instrumental variable strength

All F values of the IVs in our analyses surpassed the threshold of 10, indicating sufficient instrument strength. The Steiger filtering approach revealed that all IVs had the correct causal direction. The SNPs that were possibly related to confounding factors were removed using LDtraits tools (Table S4).

After filtering, IVs were systematically identified in both the discovery and replication analyses: 262 and 272 IVs associated with LDL-C; 301 and 314 IVs associated with TG; 21 and 20 IVs associated with Lp(a) in the European population; 27 and 24 IVs associated with LDL-C; 19 and 19 IVs associated with TG; and 1 and 1 IVs associated with Lp(a) in the East Asian population. For drug target genes, 28 and 30 IVs for *HMGCR*, 42 and 43 IVs for *PCSK9*, 13 and 13 IVs for *NPC1L1*, 25 and 25 IVs for *ANGPTL3*, 40 and 42 IVs for *APOC3*, and 63 and 66 IVs for *LPA* were available in the European population. In the East Asian population, 7 and 9 IVs for *HMGCR*, 10 and 11 IVs for *PCSK9*, 2 and 6 IVs for *ANGPTL3*, 14 and 18 IVs for *APOC3*, and 4 and 7 IVs for *LPA* were available. No IVs were available for *NPC1L1* in the East Asian population, and as a result *NPC1L1* was excluded from further analysis. The IVs for genetically proxied lipid levels and lipid-modifying drug target genes are presented in Tables S5-S6.The outcomes of the positive control study demonstrated the most significant positive associations, with the exception of the drug target gene *ANGPTL3* in the East Asian population (Table S4). The IVs of the drug target gene *ANGPTL3* were subsequently excluded from further analyses.

### Lipids and AF risk

**Fig. 2:**
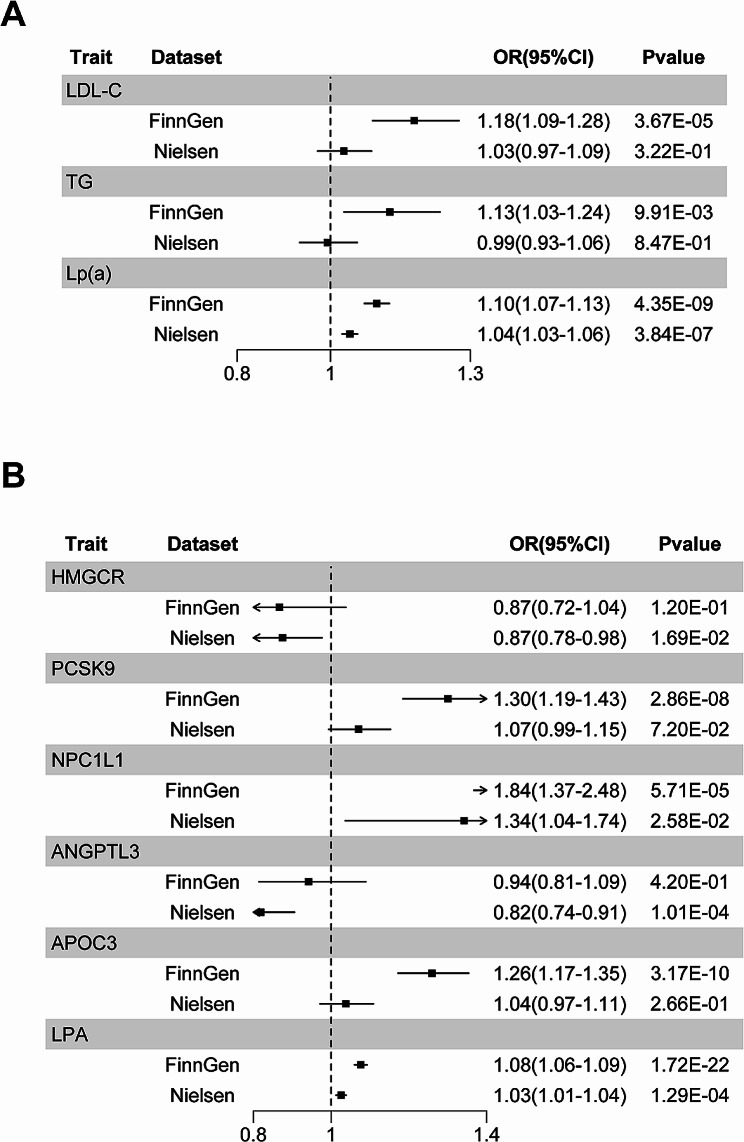
Causal effects of genetically proxied lipids (**A**) and drug target genes (**B**) on the risk of AF in the European population. The results were visualized by forest plots. Abbreviations: OR: odds ratio; CI: confidence interval; IVW: inverse variance weighted; MR: Mendelian randomization

**Fig. 3:**
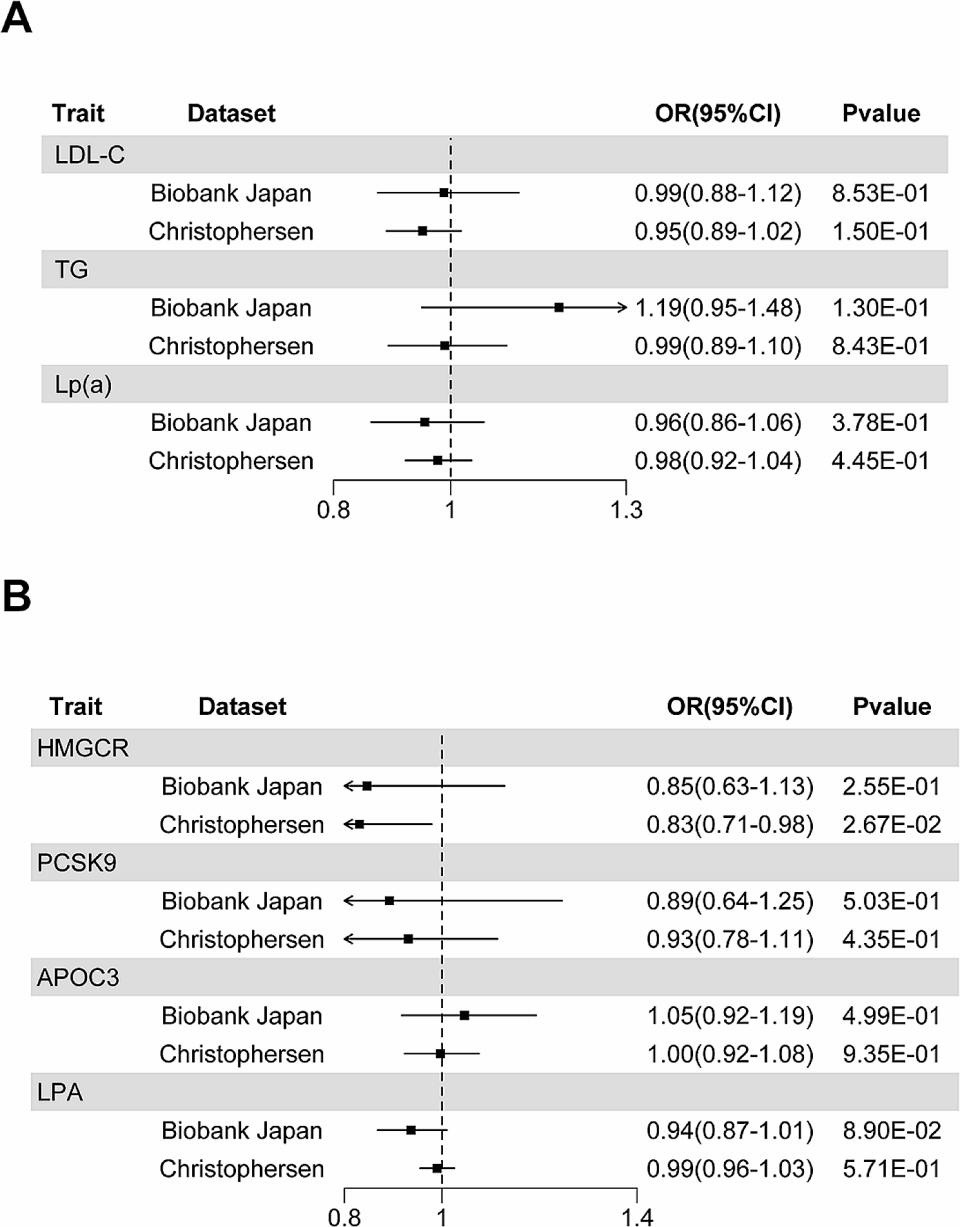
Causal effects of genetically proxied lipids (**A**) and drug target genes (**B**) on the risk of AF in the East Asian population. The results were visualized by forest plots. Abbreviations: OR: odds ratio; CI: confidence interval

In the European population, there was a potential link between elevated genetically proxied Lp(a) and LDL-C levels and an increased risk of AF in the FinnGen dataset [Lp(a), OR: 1.10, 95% confidence interval (CI): 1.07–1.13, *P =* 4.35 × 10^− 9^; LDL-C, OR: 1.18, 95% CI: 1.09–1.28, *P =* 3.67 × 10^− 5^] (Fig. 2A and Table S8). However, only the association between Lp(a) and AF was detected in the Nielsen dataset (OR: 1.04, 95% CI: 1.03–1.06, *P =* 3.84 × 10^− 7^) (Fig. 2A and Table S8). There were no statistically significant associations observed for other traits. The effect sizes from the maximum likelihood and weighted median methods followed the same trend as the IVW method. Only the exposure Lp(a) was confirmed in all results. In the East Asian population, no statistically significant correlations were detected (Fig. 3A and Table S8).

### Lipid-modifying drug target genes and AF risk

In the European population, significant associations with AF risk were found for drug targets for modifying LDL-C (*PCSK9*, OR: 1.30, 95% CI: 1.19–1.43, *P =* 2.86 × 10^− 8^; *NPC1L1*, OR: 1.84, 95% CI: 1.37–2.48, *P =* 5.69 × 10^− 5^), for modifying TG (*APOC3*, OR: 1.26, 95% CI: 1.17–1.35, *P =* 3.17 × 10^− 10^), and for modifying Lp(a) (*LPA*, OR: 1.08, 95% CI:1.06–1.09, *P =* 1.72 × 10^− 22^) (Fig. 2B and Table S9). Only the result of the drug target gene *LPA* was validated in the Nielsen dataset (OR: 1.03, 95% CI: 1.01–1.04, *P =* 3.52 × 10^− 6^) (Fig. 2B and Table S9), indicating that disrupting *LPA* expression may lower the risk of AF. There were no statistically significant associations observed for other traits. The effect sizes from the maximum likelihood and weighted median methods followed the same trend as the IVW method. Only the exposure *LPA* was confirmed in all results. In the East Asian population, there were no statistically significant connections or relationships (Fig. 3B and Table S9). The statistical powers of the MR studies are detailed in Table S10.

### Colocalization analyses

For Bayesian colocalization using GWASs of the European population, the posterior probability (H4) between Lp(a) and AF in the *LPA* gene region was 0.99. In the replication dataset, the posterior probability was 0.96. The results suggested strong evidence for colocalization and revealed that the effect was not influenced by bias from variants in LD (Table S11). The posterior probabilities between LDL-C and AF in the *PCSK9* and *APOC3* gene regions were 0.92 and 0.94, respectively, but this difference was not detected in the replication dataset. We did not find evidence of colocalization for other drug targets. Bayesian colocalization using GWASs of the East Asian population also failed to provide significant evidence. The results obtained from the cML-MA method lend further support to the significant associations between the drug target gene *LPA* and the risk of AF (theta = 0.0735, *P* = 1.79 × 10^− 22^), taking into account the possibility of horizontal pleiotropy (Table S12).

### Sensitivity analyses

The final MR model was selected on the basis of the heterogeneity test results (Table S13). The MR‒Egger intercept test revealed no evidence of pleiotropy, enhancing the reliability of causal inferences (Table S13). For the Lp(a) and Lp(a)-modifying drug target gene *LPA*, the leave-one-out analysis demonstrated that our findings were not heavily influenced by individual data points. The results further strengthened the reliability of our findings (Figure S1-S2). The scatter plot revealed the similar results computed via the IVW method, the maximum likelihood method, the weighted median method, and the MR‒Egger method, as evidenced by the slope of the line (Figure S3-S4).

### Supplementary analyses

**Fig. 4:**
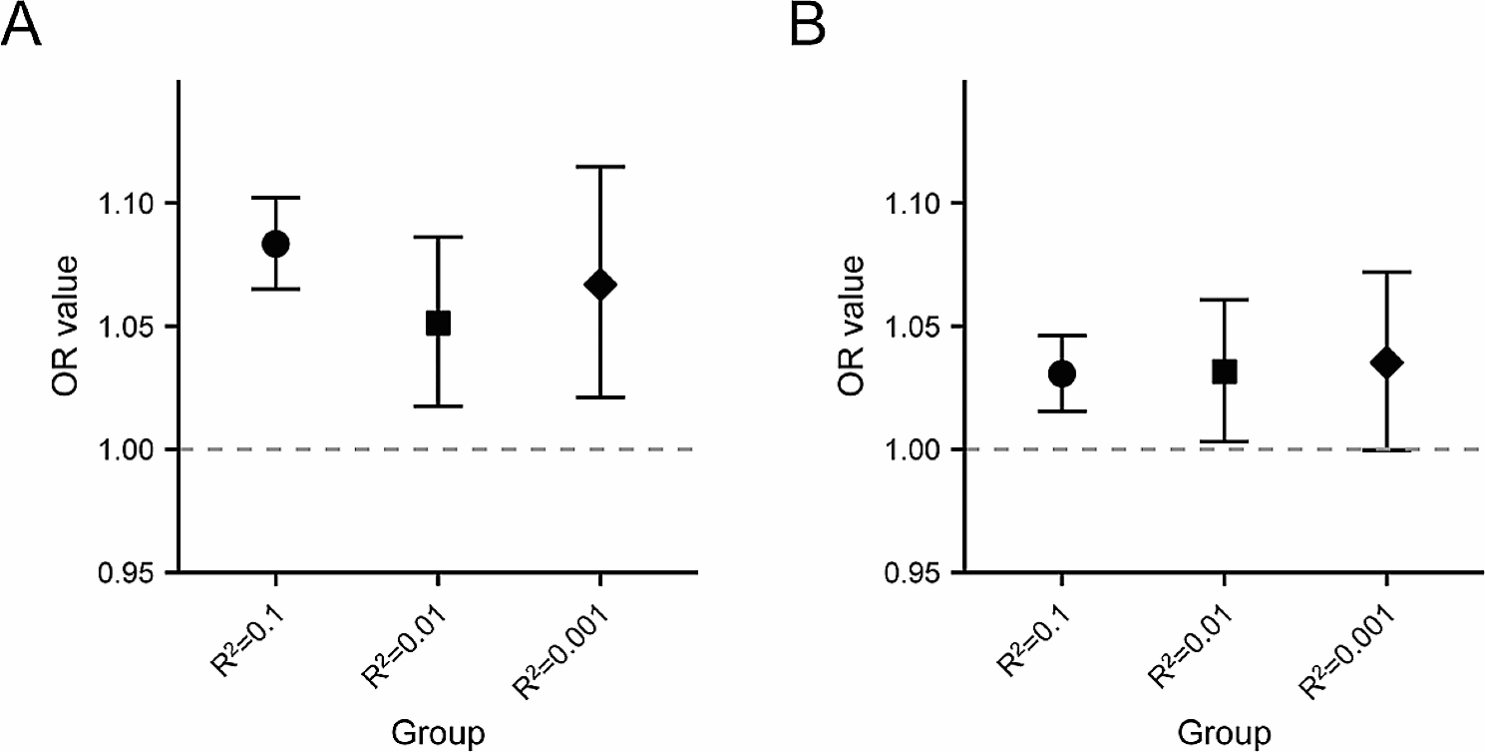
Associations of target genes under increasingly liberal LD-clumping thresholds in discovery (**A**) and validation (**B**) datasets

We performed further analyses with more stringent LD thresholds for the IVs selection of genetically proxied Lp(a)-modifying drug targets. Changes in the LD thresholds had little impact on the final results (Fig. 4 and Table S14).

SMR using cis-eQTL variants demonstrated that inhibiting the expression of *LPA* in the liver may have a protective effect against AF (OR: 1.08, 95% CI: 1.02–1.15, *P =* 1.50 × 10^− 2^). Similar trends were observed in the replication dataset, but the association did not reach statistical significance (OR: 1.01, 95% CI: 0.97–1.05, *P =* 5.58 × 10^− 1^). No significant associations were detected in the other tissues. (Table S15).

**Fig. 5:**
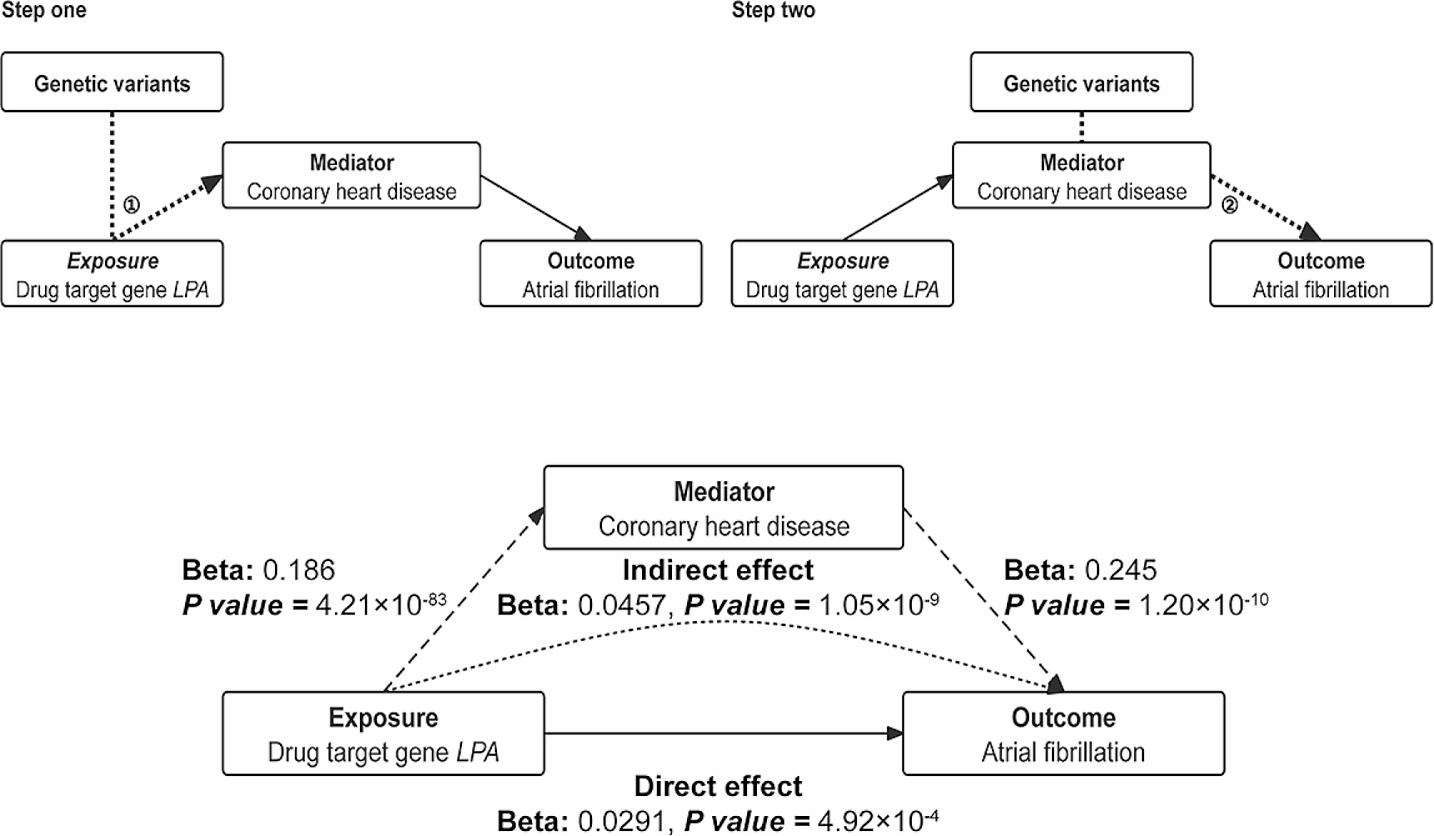
A two-step mediation analysis of the effect of the drug target *LPA* on atrial fibrillation via CHD. Abbreviations: CHD: coronary heart disease

Two-step mediation analyses revealed that the decrease in the incidence of AF resulting from *LPA* inhibition was partially mediated by the mitigation of CHD risk. The mediating effect through CHD was estimated at 0.0457 (*P =* 1.05 × 10^− 9^) (Fig. 5). Upon controlling for CHD, the direct effect was 0.0291 (*P =* 4.92 × 10^− 4^) (Fig. 5). In the mtCOJO analysis, the directionality and statistical significance of the associations remained similar to our previous findings with a reduction in the effect size (conditional analysis: beta, 3.12 × 10^− 2^, *P* = 7.02 × 10^− 5^). Conditioning on CHD, the Lp(a)-modifying gene *LPA* was still an independent factor for AF.

## Discussion

Our investigation offers compelling genetic evidence that Lp(a) is considered as a potential causal factor of AF and that the inhibition of *LPA* may contribute to reducing the risk of AF in the European population. Data on *LPA* expression in liver tissue provided additional support for this discovery. However, there remains insufficient evidence to substantiate the impact of lipids in the East Asian population.

Lp(a) refers to lipoprotein(a), which is a type of lipoprotein particle in the blood. It comprises an apolipoprotein B100 particle encapsulating cholesterol and triglycerides, intricately bound through covalent linkage with an apolipoprotein(a) unit. Observational, GWAS, and MR evidence has shown that Lp(a) is a notable risk factor for cardio-cerebrovascular diseases [43]. Lp(a) reduction has been a target for therapeutic interventions aimed at reducing cardiovascular risk [44].

Although the precise correlation between Lp(a) and AF has not been fully elucidated, several studies have explored the interconnection between Lp(a) and AF. A prior MR analysis demonstrated a significant positive association between Lp(a) levels and the risk of AF. A multivariable MR study revealed that the genetically proxied Lp(a) had a positive causal influence on the risk of AF [45]. Likewise, Mohammadi-Shemirani and colleagues, in their analysis of the UK Biobank data, identified a noteworthy correlation between Lp(a) levels and the risk of AF [46]. In a meta-analysis, it was observed that an elevation in Lp(a) levels was linked to a greater risk of AF in MR studies involving individuals of European ancestry [47]. The results of the epidemiologic study were in accordance with those of previous analysis. Li et al. demonstrated, based on observed data, that Chinese patients with AF exhibited elevated Lp(a) levels compared to those without AF. Furthermore, they established Lp(a) as an independent predictor for the onset of new AF [48]. Subsequent studies with UK Biobank data reported similar results [46]. The results suggested that the effect of Lp(a) extended across myocardial tissues. In summary, both epidemiologic and genetic analyses have provided robust support for the involvement of Lp(a) in the onset of AF. However, some findings have indicated a lack of substantial associations [49] or even presented paradoxical relationships [50, 51]. They found an inverse relationship between Lp(a) and AF, as identified through previous observational research. The findings of these studies were often influenced by various factors, such as confounding variables and the potential for reverse causation. However, our MR analysis offered clearer insight into a causal link between the two, effectively mitigating the biases associated with confounding factors and reverse causation. Our analysis could therefore add depth to the understanding of this relationship.

The concentration of Lp(a) in plasma is mainly determined by genetics. Apolipoprotein(a), encoded by the *LPA* gene, is responsible for the distinctive structure and function of Lp(a) in the bloodstream [52, 53]. At present, however, no direct therapies for lowering Lp(a) have been approved [44]. One promising approach to reduce Lp(a) levels is by inhibiting *LPA* expression with targeted drugs. Three potential therapeutic agents that reduce Lp(a) levels directly are currently in different stages of development. These drugs utilize small interfering RNAs, namely olpasiran and SLN360, or use antisense oligonucleotide technology, as seen with pelacarsen [44, 54]. They are designed with liver-targeted delivery, which is achieved by an N-acetylgalactosamine conjugate. Recently, an orally administered small molecule inhibitor of Lp(a) called Muvalaplin completed phase I clinical trial [55]. All of these agents have been proven to be effective at reducing Lp(a) concentrations and are considered beneficial and safe for CHD treatment in clinical trials [56–58]. There are few studies on the effect of lipid drugs on the risk of AF. It has been suggested that lipid-lowering drugs, such as statins, could offer benefits in preventing AF only in high-risk populations [8, 59]. In addition, there is still a lack of studies on new drugs targeting *LPA* gene expression in AF. It remains uncertain whether reducing Lp(a) concentrations with these new therapies will mitigate the risk of AF. Our findings suggest the inclusion of AF as an additional endpoint in prospective research on targeted drugs for Lp(a).

In our subsequent mediation analyses, we opted to include CHD as a possible mediating factor. Elevated Lp(a) levels are a known factor contributing to CHD, and significant reductions in Lp(a) levels may indeed manifest a clinically meaningful decrease in the risk of CHD [60]. Patients with CHD are more prone to developing AF, particularly new-onset AF [61]. The pathophysiological mechanisms of AF include re-entrant circuits, focal ectopic activity, and neural remodeling, all of which can be induced by CHD [61]. The mediation analyses revealed that the effect of the Lp(a)-modifying drug gene *LPA* on AF was partially mediated by CHD. Inhibiting Lp(a)-modifying drug target could prevent AF in patients with CHD. Besides, conditioning on CHD, the Lp(a)-modifying gene *LPA* was still an independent factor for AF. The potential mechanism of the drug extended beyond its anti-atherosclerotic effects.

Several proposed mechanisms have been proposed to account for this direct effect. Inflammation is known to have a substantial influence on both the initiation and maintenance of AF. Additionally, inflammatory processes can exacerbate oxidative stress, instigate fibrotic changes, and inflict damage upon the myocardium, thus significantly augmenting their collective contribution to the pathogenesis of AF [62]. Lp(a) possesses proinflammatory properties that affect atrial remodeling and electrical signaling [46]. Oxidized phospholipids were essential for driving this process. Oxidized phospholipids preferentially bind to Lp(a) and fuel further inflammation [63]. Direct Lp(a)-lowering therapy may have beneficial effects on inflammation and immune system regulation, including the induction of anti-inflammatory gene expression and decreased activation of circulating monocytes [64]. Oxidized phospholipids serve as the signaling molecules for monocyte activation via Toll-like receptor recognition. Previous studies have demonstrated that genes associated with the Toll-like receptor pathway exhibit upregulation in response to elevated Lp(a) levels, and subsequently undergo downregulation following targeted reduction of Lp(a) through therapeutic intervention.

Previous MR analyses were only conducted using a two-sample method in the European population. No further analysis was performed to validate the results. Our study is the first MR study using different methods to assess the causal effect of Lp(a) and the drug target gene *LPA* on AF. To obtain reliable conclusions, we conducted a comprehensive and robust evaluation of the results. We utilized a larger set of genetic instruments identified from the GWASs. Our colocalization analysis was essential because it provided valuable complementary information, indicating that our findings are less likely to be explained by LDs. Moreover, we incorporated additional evidence from gene expression data derived from liver tissue data to reinforce the findings from a different point of view. In addition, we searched for one of the most confounding factors, CHD status, and then evaluated its effect to reduce the bias. We conducted mediation analysis to further assess the indirect effect. Also, we offered different insights by using data from European and East Asian ethnical groups.

However, there are some limitations in our study. First, it is impossible to equate the effects of genetically proxied drug targets with those observed in RCTs. Estimates from MR analyses represent lifelong effects, while drugs are usually taken for a specific duration. MR analyses typically prioritize the direction of associations over quantitative estimates. Nevertheless, our conclusions may offer valuable insights into AF prevention, supporting the importance of further clinical trials for validation.Second, the findings may have been affected by the limited statistical power due to the relatively small sample size of exposures and outcomes. The available GWASs for East Asian populations are currently limited in number, which in turn leads to less reliable conclusions. Third, sufficient SNPs are required in the HEIDI test, and we could not obtain enough SNPs for use in the test. Only the *LPA* eQTLs in the esophageal mucosa showed enough IVs. Neither the SMR nor the HEIDI test yielded significant results. We will validate our results for these tests once a sufficient sample of data becomes available. Fourth, stratification of the primary GWAS datasets based on specific subtypes is lacking. Consequently, the execution of a stratified analysis proved unfeasible within the confines of the current investigation. This area warrants further scholarly exploration. In addition, horizontal pleiotropy, as an inherent limitation of MR analysis, is difficult to completely avoid, although we conducted several sensitivity analyses and tried to best minimize the impact of pleiotropy.Finally, the study population included only participants of European and East Asian ancestry. Certain investigations have indicated that the plasma concentrations of Lp(a) exhibit a pronounced reliance on ethnicity. To assess the generalizability of our findings, future studies among other ethnic populations are necessary.

## Conclusion

In summary, our research provides insights into the potential role of Lp(a) in the risk profile of AF in the European population. Our results suggest that higher genetically proxied Lp(a) levels may be associated with an increased propensity for the development of AF. Furthermore, our research revealed a possible protective effect of inhibiting the LPA gene. This effect occurs partially through a mechanism involving lowering the risk of CHD. Our findings play a crucial role in addressing the existing gap in preventive treatments for AF, and underscore the potential of *LPA* as a promising target for pharmacological intervention. Further mechanistic research and better powered epidemiologic studies are needed to examine this association.

### Electronic supplementary material

Below is the link to the electronic supplementary material.

Supplementary Material 1

Supplementary Material 2

## Data Availability

No datasets were generated or analysed during the current study.

## Abbreviations

AF: atrial fibrillation
LDL-C: Low-density lipoprotein cholesterol
TG: Triglyceride
Lp(a): lipoprotein(a)
CHD: coronary heart disease
RCTs: randomized controlled trials
MR: mendelian randomization
IV: instrumental variables
SNPs: single nucleotide polymorphisms
GWAS: genome-wide association studies
eQTL: expression quantitative trait loci
LD: linkage disequilibrium
GTEx: Genotype-Tissue Expression
IVW: inverse variance weighted
SMR: summary-databased mendelian randomization
cML-MA: constrained maximum likelihood and model averaging
OR: odds ratio
CI: confidence interval
HEIDI: heterogeneity in dependent instruments
