# Supplementary material for: Associations of lipids and lipid-modifying drug target genes with atrial fibrillation risk based on genomic data
Source: Lipids Health Dis. 2024 Jun 8;23:175. doi: 10.1186/s12944-024-02163-4 (PMC11161942; doi:10.1186/s12944-024-02163-4)

**Supplementary Figure Legends**

**Supplementary Figure S1.** Leave-one-out plots for the lipoprotein(a) on atrial fibrillation. The results of the datasets of (A) FinnGen or (B) Nielsen.

**Supplementary Figure S2.** Leave-one-out plots for the drug target *LPA* on atrial fibrillation in the datasets of (A) FinnGen or (B) Nielsen.

**Supplementary Figure S3.** Scatter plots of the genetic associations between the lipoprotein(a) and atrial fibrillation in the datasets of (A) FinnGen or (B) Nielsen.

**Supplementary Figure S4.** Scatter plots of the genetic associations between the drug target *LPA* and atrial fibrillation in the datasets of (A) FinnGen or (B) Nielsen.

**Supplementary Figure S1.**


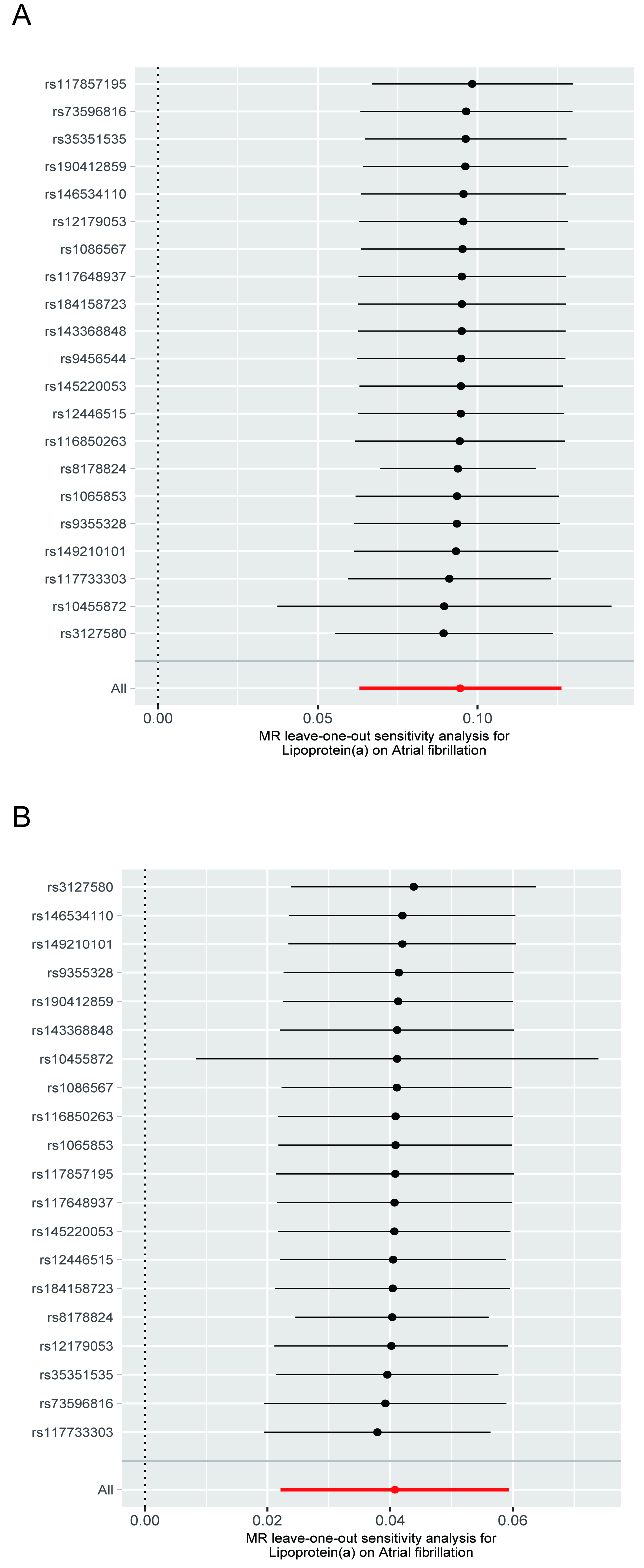


**Supplementary Figure S2.**


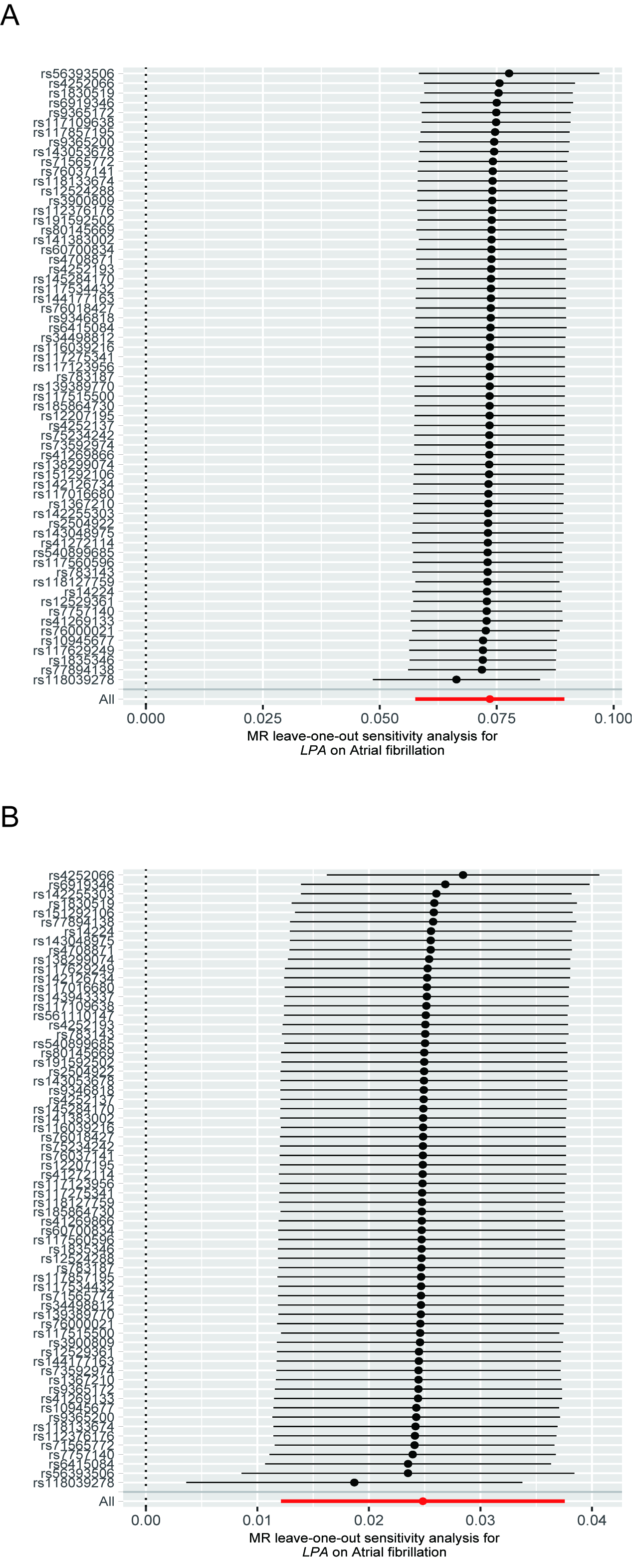


**Supplementary Figure S3.**


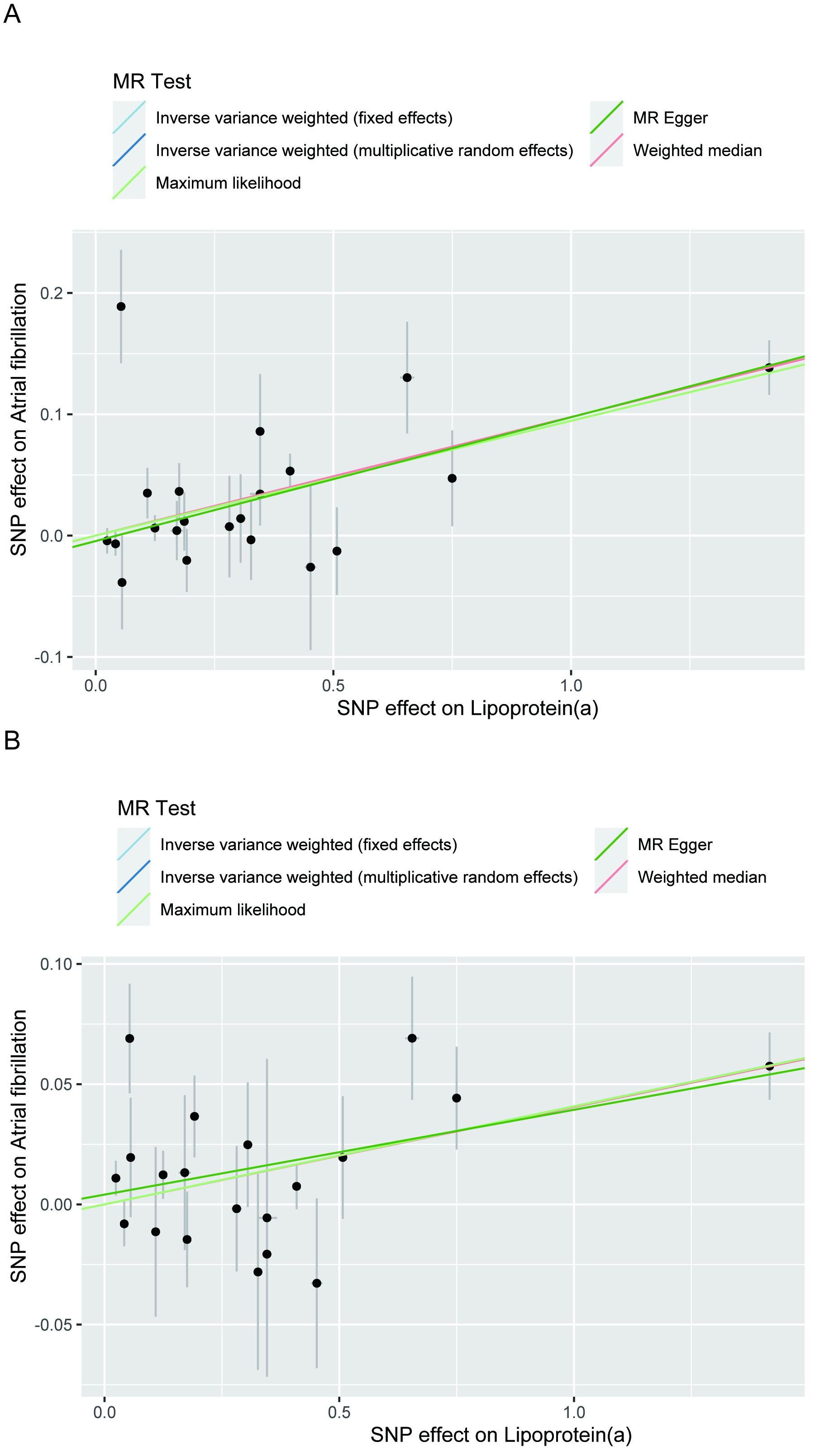


**Supplementary Figure S4.**


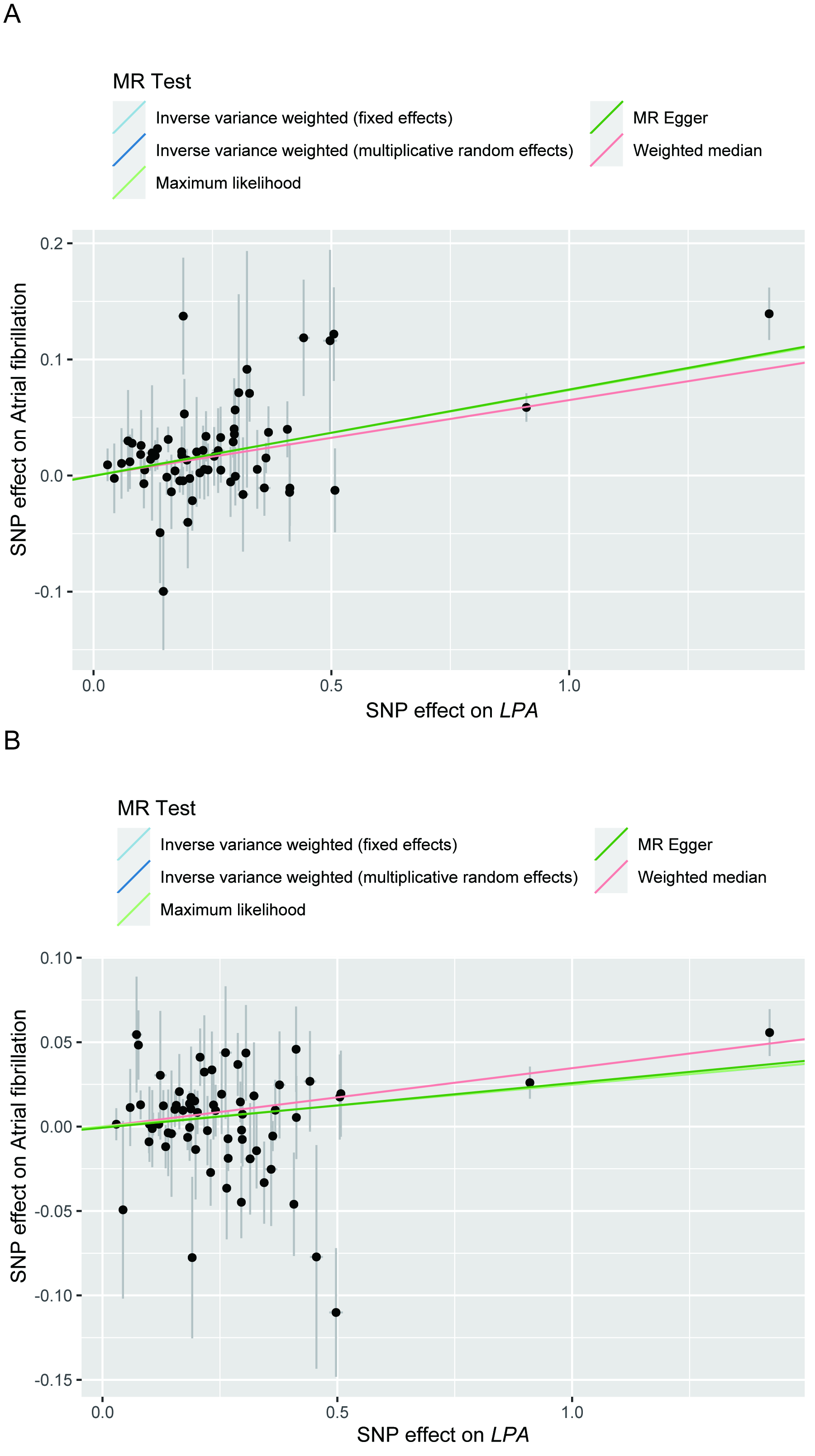

Supplement: Supplementary file 1 — Supplementary Material 1 [file 12944_2024_2163_MOESM1_ESM.docx]
